# Theoretical study of the Usutu virus helicase 3D structure, by means of computer-aided homology modelling

**DOI:** 10.1186/1742-4682-6-9

**Published:** 2009-06-25

**Authors:** Dimitrios Vlachakis

**Affiliations:** 1 Institute of Biology, National Centre for Scientific Research Demokritos, 15310 Ag. Paraskevi Attikis, Greece

## Abstract

**Background:**

Usutu virus belongs to the *Flaviviridae *viral family and constitutes an important pathogen. The viral helicase is an ideal target for inhibitor design, since this enzyme is essential for the survival, proliferation and transmission of the virus.

**Results:**

Towards a drug-design approach, the 3D model of the Usutu virus helicase structure has been designed, using conventional homology modelling techniques and the known 3D-structure of the Murray Valley Encephalitis virus helicase, of the same viral family, as template. The model was then subjected to extended molecular dynamics simulations in a periodic box, filled with explicit water molecules for 10 nanoseconds. The reliability of the model was confirmed by obtaining acceptable scores from a variety of *in silico *scoring tools, including Procheck and Verify3D.

**Conlcusion:**

The 3D model of the Usutu virus helicase exhibits *in silico *all known structural characteristics of the *Flaviviridae *viral family helicase enzymes and could provide the platform for further *de novo *structure-based design of novel anti-Usutu agents.

## Background

The viral family *Flaviviridae *comprises the genera *Flavivirus, Pestivirus *and *Hepacivirus *and includes numerous important human and animal pathogens [1]. The small, enveloped virions of the different members of the *Flaviviridae *family contain a single-stranded, positive-sense RNA genome of about 9.5–12.5 kb. The genome consists of a single, long open reading frame (ORF), which is flanked by untranslated regions (UTRs) at the 5' and 3' ends.

Recent studies on sub-genomic *Pestivirus *and *Flavivirus *RNA replicons have revealed that the non-structural (NS) proteins, which are encoded by the C-terminal part of the polyprotein, play a crucial role in viral RNA replication [2,3]. Accordingly, these proteins are assumed to form replication complexes in conjunction with genomic RNA and possibly with other cellular factors.

Sequence alignments of the Usutu viral helicase identified several conserved sequence motifs that are important for biological functions. So far, the crystal structures of helicases from various RNA viruses have been determined, including the helicases from hepatitis C virus, Dengue virus, Yellow Fever Virus and Kunjin virus [4].

Usutu virus is the cause of one of the most important arthropod-borne viral (arbovirus) diseases, transmitted mainly by the *Culex pipiens *mosquito [5]. Usutu virus was first detected in Africa, and four decades later it emerged in Austria. Usutu virus is closely related to the more common West Nile virus, Japanese Encephalitis virus and Yellow Fever virus. Usutu virus caused mass mortalities of birds, especially blackbirds (*Turdus merula*) and great grey owls (*Strix nebulosa*) initially in Austria, but then spread to other central European countries such as Hungary, Switzerland, and Northern Italy (in chronological order) [6].

In the present work, the three-dimensional structure of the helicase enzyme of Usutu virus was modelled by applying homology modelling techniques and using the crystal structure of the Murray Valley Encephalitis virus helicase of the *Flaviviridae *family as template.

## Methods

### Sequence alignment

The amino acid sequence of Usutu viral helicase was obtained from the GenBank database (accession no.: NC_006551, entry name: Usutu virus, complete genome). Using the Gapped-BLAST [7] through NCBI [8]. the homologous structure of Murray Valley Encephalitis virus helicase was identified, which was used as template for the homology modelling of the Usutu viral helicase. The sequence alignment was done using the online version of ClustalW [9].

### Secondary structure prediction

Secondary structure predictions were performed using the NPS (Network Protein Sequence Analysis) web-server  and the GeneSilico MetaServer  in order to confirm that the chosen Murray Valley Encephalitis virus helicase template is the most appropriate one [10].

### Homology modelling

The homology modelling of the Usutu viral helicase was carried out using the MOE (Molecular Operating Environment, version: 2004.03) package and its built-in homology modelling application [11]. The RCSB entry 2V80, which corresponds to the crystal structure of the Murray Valley Encephalitis virus helicase was used as template. The sequence alignment between the raw sequence of the Usutu and the sequence of the Murray Valley Encephalitis virus template revealed almost 90% identity, which allows for a conventional homology modelling approach to be considered. The homology model method of MOE comprises the following steps: First an initial partial geometry specification, where an initial partial geometry for each target sequence is copied from regions of one or more template chains. Secondly, the insertions and deletions task, where residues that still have no assigned backbone coordinates are modelled. Those residues may be in loops (insertions in the model with respect to the template), they may be outgaps (residues in a model sequence which are aligned before the C-terminus or after the N-terminus of its template) or may be deletions (regions where the template has an insertion with respect to the model). For this study though outgaps have not been included in the homology modelling process. Third step is the loop selection and sidechain packing, where a collection of independent models is created. Last step is the final model selection and refinement one, where the final models are scored and ranked, after they have been stereochemically tested and evaluated with the built-in module "Protein Geometry" for errors.

### Molecular electrostatic potential (MEP)

Electrostatic potential surfaces were calculated by solving the nonlinear Poisson-Boltzmann equation using finite difference method as implemented in the PyMOL Software [12]. The potential was calculated on grid points per side (65, 65, 65) and the grid fill by solute parameter was set to 80%. The dielectric constants of the solvent and the solute were set to 80.0 and 2.0, respectively. An ionic exclusion radius of 2.0Å, a solvent radius of 1.4Å and a solvent ionic strength of 0.145 M were applied. Amber99 [13] charges and atomic radii were used for this calculation.

### Model optimization

Energy minimisation was done in MOE initially using the Amber99 [13] forcefield implemented into the same package, up to a RMSd gradient of 0.0001 to remove the geometrical strain. The model was subsequently solvated with SPC water using the truncated octahedron box extending to 7Å from the model and molecular dynamics were performed after that at 300 K, 1 atm with 2 fsecond step size and for a total of ten nanoseconds, using the NVT ensemble in a canonical environment. NVT stands for Number of atoms, Volume and Temperature that remain constant throughout the calculation. The results of the molecular dynamics simulation were collected into a database by MOE and can be further analysed.

### Model evaluation

The produced models were initially evaluated within the MOE package by a residue packing quality function, which depends on the number of buried non-polar side chain groups and on hydrogen bonding. Moreover, the suite PROCHECK [14] was employed to further evaluate the quality of the produced Usutu virus helicase model. Finally, Verify3D [15,16] was used to evaluate whether the model of Usutu virus helicase is similar to known protein structures.

## Results and discussion

**Figure 1:**
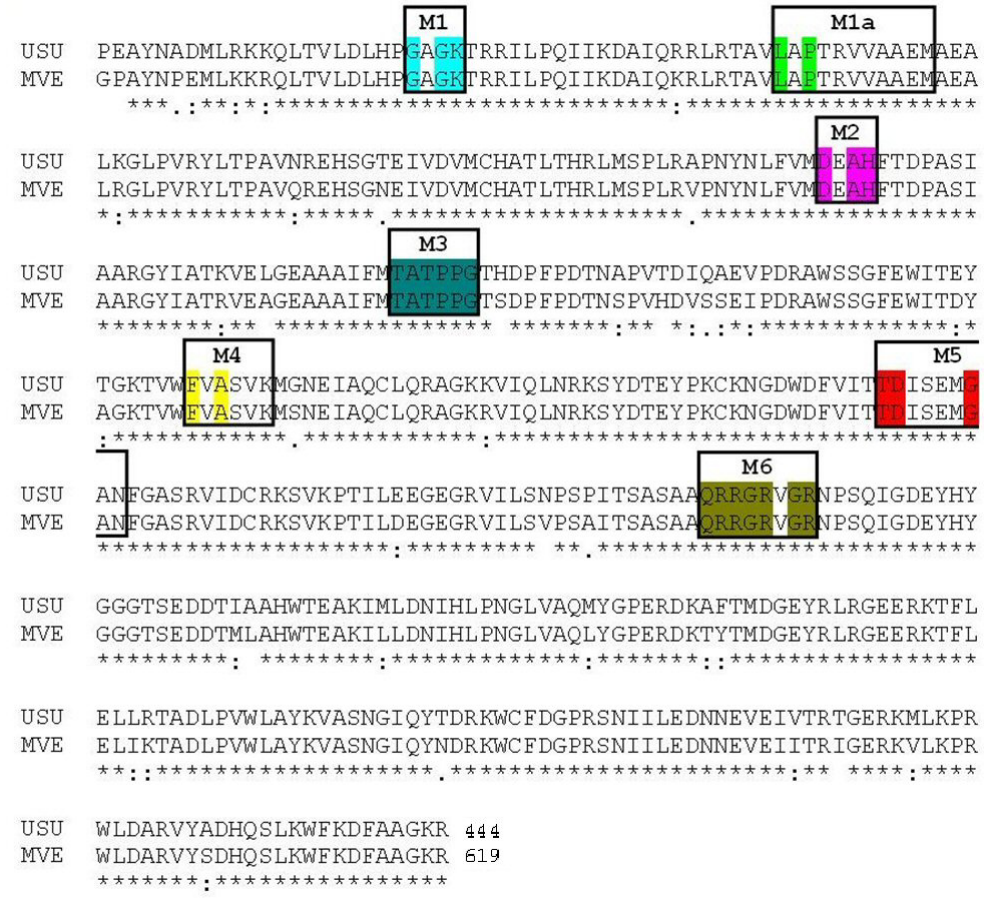
**The sequence alignment between the Usutu virus helicase sequence and the corresponding sequence of the Murray Valley Encephalitis virus helicase template (RCSB entry: 2V80)**. All seven major conserved motifs of *Flaviviridae *helicases have been highlighted.

The NS3 domain of *Flaviviridae *contains both the protease and the helicase coding regions. For this study, the Usutu virus helicase sequence was aligned with the Murray Valley Encephalitis virus helicase template sequence and all the major helicase motifs, characteristic of the *Flaviviridae *family were found to be conserved (Figure 1).

The Murray Valley Encephalitis virus helicase structure has been established by X-ray crystallography at 1.9 Å resolution [17]. The Murray Valley Encephalitis virus helicase is an appropriate template because the virus belongs to the same viral family (*Flaviviridae*). Among the available helicase structureswithin this family, the Murray Valley Encephalitis virus enzyme presented the highest identity percentage to the Usutu virus helicase. That is also supported by the analysis of the helicase structures available for the whole *Flaviviridae *family that revealed that the overall 3D structure for the Murray Valley Encephalitis virus helicase ishighly conserved [17,18]. Moreover using a fold recognition approach (FR), the Murray Valley Encephalitis virus helicase was confirmed as the most appropriate model. The secondary structure prediction for the Usutu virus helicase turned out to be very similar to the actual structure of the Murray Valley Encephalitis virus helicase. Methods of protein fold recognition attempt to detect similarities between protein 3D structure that are not accompanied by any significant sequence similarity. There are many approaches, but the unifying theme is to try and find folds that are compatible with a particular sequence. Unlike sequence-only comparison, these methods take advantage of the extra information made available by 3D structure information. In effect, the turn the protein folding problem on it's head: rather than predicting how a sequence will fold, they predict how well a fold will fit a sequence [19-22].

The model was firstly structurally superimposed and subsequently compared to its template, where exhibited an alpha-carbon RMSD less than 0,65 angstroms. Furthermore it was evaluated with MOE and PROCHECK for its geometry [see additional file 1] and then subjected to the Verify3D algorithm for a more thorough evaluation. Verify3D assessed the compatibility of the 3D model of Usutu virus helicase with its own amino acid sequence. Based on location and environment, a structural class is assigned for each residue. A collection of reference structures is used as a control in order to calculate a score for each residue. The Usutu virus helicase model ranged from +0.32 to +0.84. That confirmed that the model is of high quality, since it is Verify3D scores below +0.1 that are indicative of serious problems in the model [23]. A more important criterion was the compliance of the model with the known unique characteristics of the helicases that belong to the *Flaviviridae *family of viruses. The Murray Valley Encephalitis virus, Hepatitis C virus, Dengue virus, West Nile virus and Usutu virus helicases all belong to the superfamily II of helicases and share seven common motifs within their domains, all of which have been structurally conserved on the Usutu virus helicase model [17].

### Description of the Usutu virus helicase model

**Figure 2:**
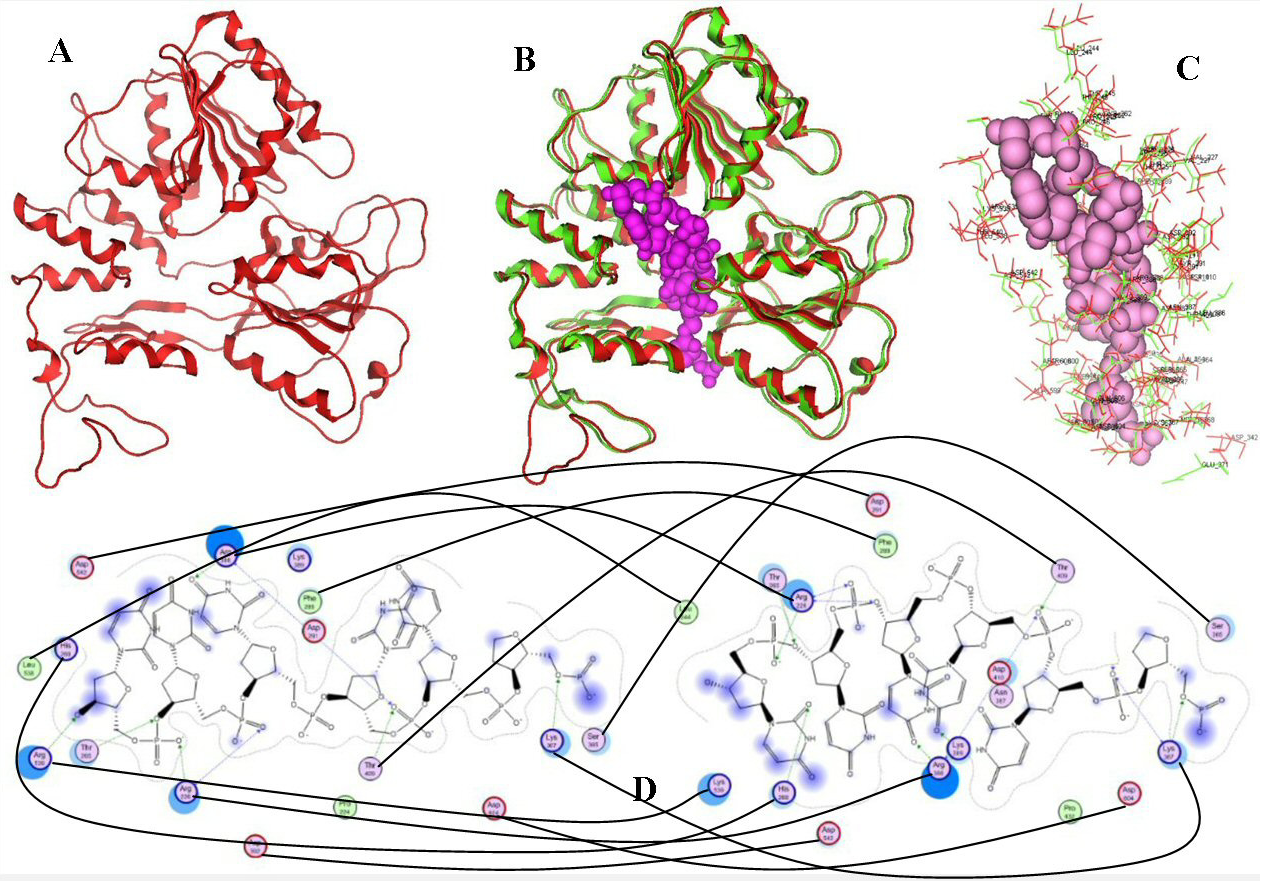
**A: Ribbon representation of the produced Usutu virus helicase model**. **B**: the Usutu virus helicase model superimposed with its Murray Valley Encephalitis virus helicase template (RCSB entry: 2V80). The Usutu virus helicase model is in red, whereas the Murray Valley Encephalitis virus helicase template is in green and the ssRNA substrate (coordinates obtained from 1A1V [24]) is in magenta. **C**: All residues surrounding the ssRNA fragment, color-coded as B. The ssRNA interacting regions are almost identical between the Usutu virus helicase model and the MVE helicase template. **D**: The interaction map of the per-residue ssRNA interaction pattern from Ligplot for Usutu virus helicase model (left) and the Murray Valley Encephalitis virus helicase template (right). Conserved residues that are involved in the ssRNA binding and are common between the Usutu virus helicase model and the MVE helicase template have been connected with a line.

**Figure 3:**
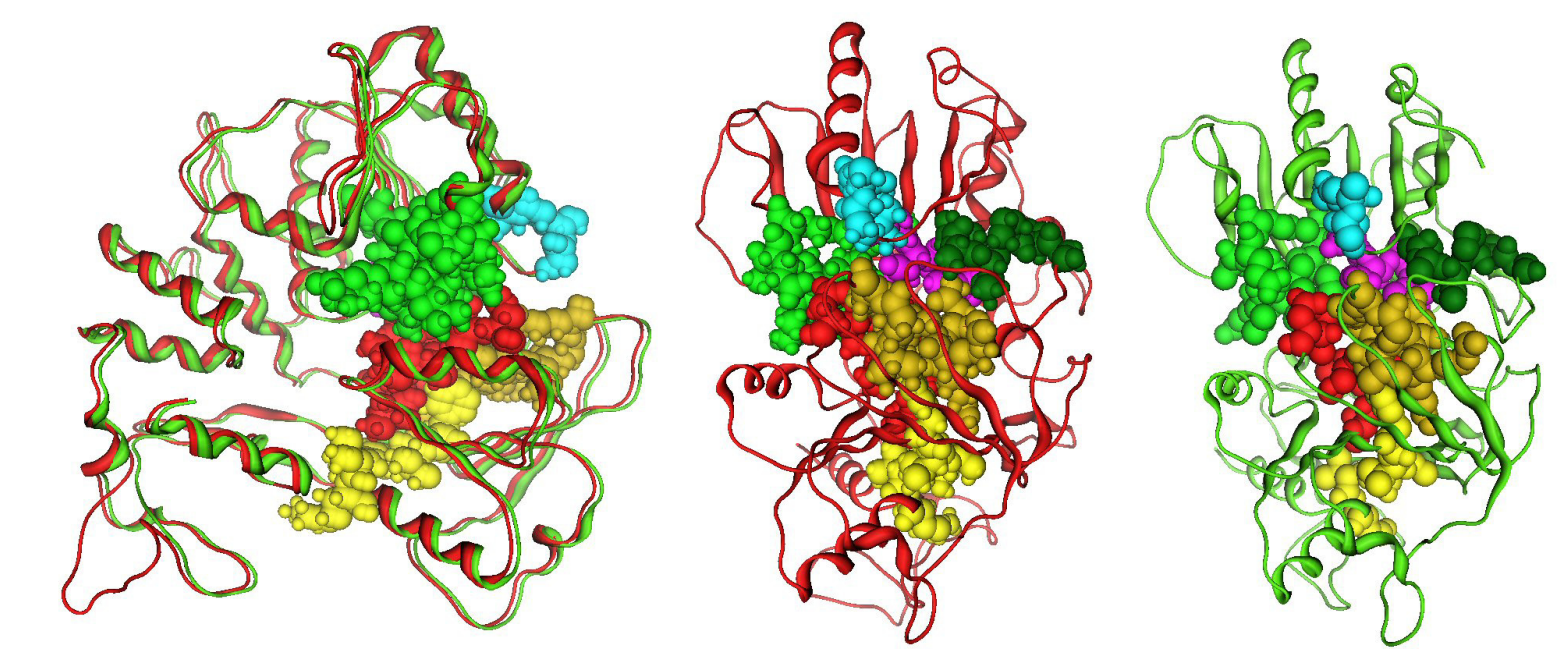
**The conserved motifs of the Murray Valley Encephalitis virus helicase (in green color) next to the corresponding motifs on the Usutu virus helicase model (colored in Red)**. The major motifs have been color-coded according to the conventions of Fig. 1, and are showing in CPK format (Usual space filling) along with the rest of the helicase motifs.

As expected from the sequence alignment (Figure 1) and the secondary structure prediction (data not shown), the Usutu virus helicase model exhibited the structural features of known *Flaviviridae *helicases. Namely, the three distinct domains of helicases as well as the various motifs (as reported by Kim [24] – Figure 1) were structurally conserved in the Usutu virus helicase model (Figures 2 and 3). One of the most crucial motifs in *Flaviviridae *helicases is the GxGKT/S motif in domain 1, which is conserved to the same loop in kinases. It is a Walker A motif and its role involves binding of the β-phosphate of ATP [25]. Site directed mutagenesis studies of that motif have reported that the mutant protein is inactive. Furthermore another crucial motif for the helicase is the DExH motif. The DExH motif is responsible for the binding of the Mg2+-ATP substrate. Studies in adenylate and thymidine kinases revealed that an aspartate (Asp170) binds the Mg2+ and helps to establish the optimum orientation of ATP for nucleophilic attack [26]. Finally, another crucial motif is the QRxGRxGR motif. The role of this motif is exceptionally crucial to the *Flaviviridae *helicase function as it is involved in nucleic acid binding [27-29].

**Figure 4:**
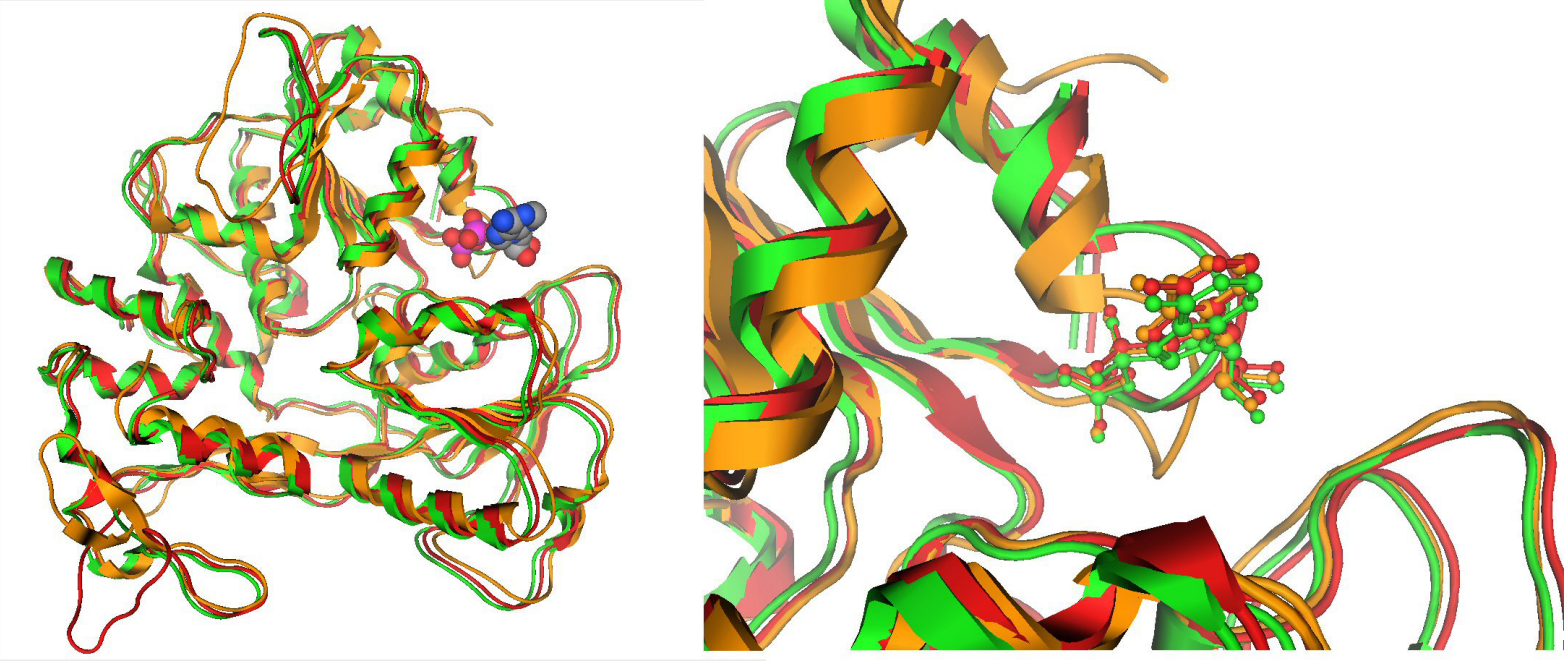
**The ATP site of the three helicase structures**. Left: The Murray Valley Encephalitis helicase is in green color, the Dengue helicase is in orange color, the Usutu helicase model is colored red and the ADP molecule, is showing in CPK format (ADP conformation adopted from the X-ray structure of the Dengue Virus helicase: 2JLS [30]). Right: Zoom-in of the ADP binding site and the conformation of the ADP molecule after the MD simulation color-coded for each receptor. The binding mode of the ADP molecule is exactly the same in the Usutu virus helicase model, as it is in the Murray Valley Encephalitis virus template structure.

**Figure 5:**
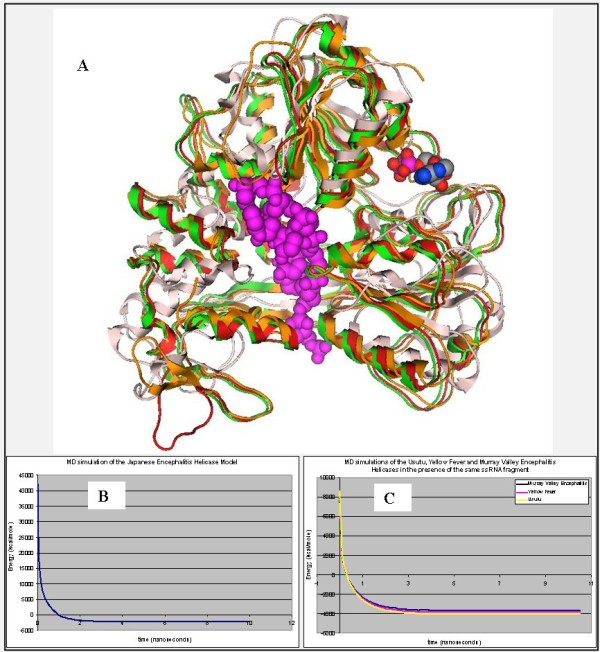
**A: The ssRNA channel site of the four helicase structures: the Murray Valley Encephalitis helicase is in green color, the Yellow fever helicase is in pink color, the Dengue helicase is in orange color and the Usutu helicase model is colored red**. The ssRNA substrate, indicating the nucleic acid binding channel is showing in magenta spacefill representation and the NTP binding site is in the proximity of the ADP molecule, showing in CPK format. **B**: The energy vs time plot of the full MD simulation of the Usutu helicase model. The 10 ns MD simulation quickly reached equilibrium and remained stable. **C**: Energy vs time plot of 10 ns MD simulation for each of the two X-ray structures (Murray Valley Encephalitis and Yellow Fever helicases) and the Usutu helicase model in the presence of the ssRNA fragment, borrowed from the Murray Valley Encephalitis structure (id: 2V80). It was demonstrated that the Usutu helicase model + ssRNA complex (yellow) behaved in a way very similar to that of the Murray Valley Encephalitis X-ray structure + ssRNA (blue) and Yellow Fever X-ray structure + ssRNA (magenta) complexes.

The model shared a very similar topology (shape and size) to its template (Figures 2 and 3). The *Flaviviridae *helicases, in general, have three domains in total, which are separated by two channels. The first and third domains are interacting much more together than they do with domain two. Domain two is supposed to undergo significant movements compared to the other two domains, during the unwinding of double-stranded nucleic acids. It is the channel between domain 3 and 1–2 that accommodates the ssRNA during the viral unwinding process. RNA binds to the helicase at the arginine-rich site of the 2^nd ^domain. The ATP and ssRNA sites were found to have been conserved on the Usutu virus helicase model (Figures 4 and 5) [30].

### ssRNA – ATP substrates and MD simulations

In order to evaluate the substrate binding site, the model was subjected to energy minimization and molecular dynamics (MD) simulations in the presence of the helicase substrates. The coordinates of the ssRNA and ADP/Mn^++ ^were transferred to the model from the Hepatitis C (1A1V) and the Dengue virus helicase structure (2JLS) respectively, for this purpose. Invariant residues of various motifs in the vicinity of either substrate in the Murray Valley Encephalitis virus template structure were conserved structurally in the Usutu virus helicase model (Figures 2 and 4). Most interactions between the ssRNA fragment and the Usutu helicase enzyme are established with the backbone of the ssRNA fragment, which is typical for non-specific protein – nucleic acid interactions. The bases in the middle of the ssRNA do not seem to interact much with the receptor (Figure 2D). The receptor's contacts emerge mostly from domains one and two of the Usutu helicase and most specifically, by loops between secondary structure elements of the latter domains. More detailed (i.e. per residue) comparison of the ssRNA interaction pattern between the Usutu virus helicase model and the Murray Valley Encephalitis virus helicase structure has been drawn using LigPlot, which is a built-in module of MOE, and is shown in Figure 2D.

The 10 ns MD simulations revealed that soon the Usutu virus helicase model reached a conformational equilibrium similar to that of Murray Valley Encephalitis virus helicase structure (Figure 5B and 5C). Taken together these observations illustrated the viability of the homology modelling of the Usutu virus helicase model. To evaluate further the Usutu virus helicase model, the latter was compared with its template structure by calculating the root mean square deviations (RMSd) between equivalent atoms for the full MD course. Large RMSd values are indicative of systems of poor quality. The C^α ^RMSd of the Usutu virus helicase model from the equivalent domains of the template structures was less than 0.65Ǻ. The low RMSd value indicated that this model remains conformationally close to the template structure upon the minimization and the molecular dynamics simulation course that followed, reflecting its high quality.

### Molecular surface analysis

**Figure 6:**
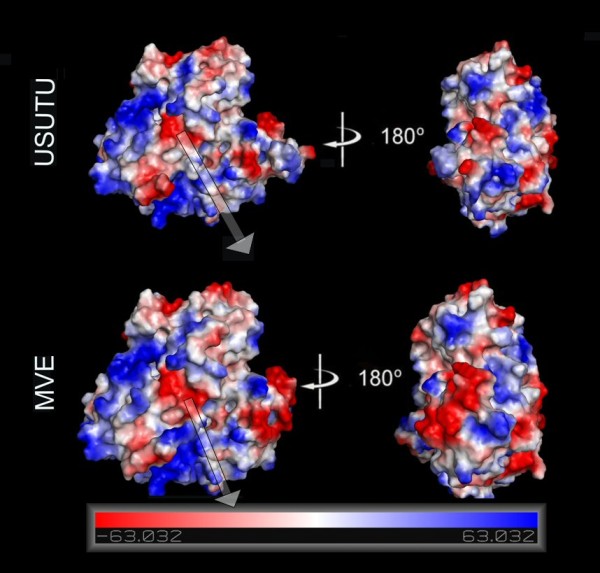
**Electrostatic surfaces potential for the Usutu virus helicase model and the Murray Valley Encephalitis virus helicase template (X-ray structure: 2V80)**. White transparent arrow is showing the nucleic acid channel (ssRNA).

In order to analyze the molecular surface of the produced Usutu virus helicase model, the electrostatic potential surface was calculated. For direct comparison with the template structures used in this study, electrostatic potential surfaces were also calculated for the Murray Valley Encephalitis virus helicase (Figure 6). The two helicases exhibited almost identical electrostatic surfaces, sharing common features such as a negatively charged ssRNA entrance to the helicase tunnel. This observation verified the validity of the model, which was found to share similar electrostatic surface to its X-ray crystal structure template.

## Conclusion

The 3D model of the Usutu virus helicase was designed using the homologous X-ray crystal structure of the Murray Valley Encephalitis viral helicase as template. The model was successfully evaluated both in terms of its geometry, fold recognition and compliance to the criteria required as a member to the *Flaviviridae *viral family. It is therefore proposed that the Usutu virus helicase model will be suitable for further *in silico *structure-based *de novo *drug design experiments. These computer-based methodologies are now becoming integral part of the drug discovery process that may eventually lead to the development of potential inhibitor structures of the Usutu virus helicase enzyme in the future.

## Competing interests

The author declares that they have no competing interests.

## Authors' contributions

DV is the sole author of this paper and is responsible for developing the concepts and for writing and revising the manuscript.

## Supplementary Material

Additional file 1 **Extended Procheck Results for both the Usutu virus helicase model and the Murray Valley Encephalitis virus helicase template**. Extended Procheck Results for both the Usutu virus helicase model (LEFT COLUMN) and the Murray Valley Encephalitis virus helicase template (RIGHT COLUMN, X-ray structure: 2V80). A: Ramachandran plot, B: Bond Length Plot, C: Bond Angles plot, D: Dihedrals plot, E: Rotamers plot and F: Contact Energies Plot. It is therefore concluded that the Usutu virus helicase model has inherited all structural characteristics of its Murray Valley Encephalitis virus helicase template. Click here for file
