# Supplementary figures and images for: Theoretical study of the Usutu virus helicase 3D structure, by means of computer-aided homology modelling
Source: Theor Biol Med Model. 2009 Jun 25;6:9. doi: 10.1186/1742-4682-6-9 (PMC2707372; doi:10.1186/1742-4682-6-9)

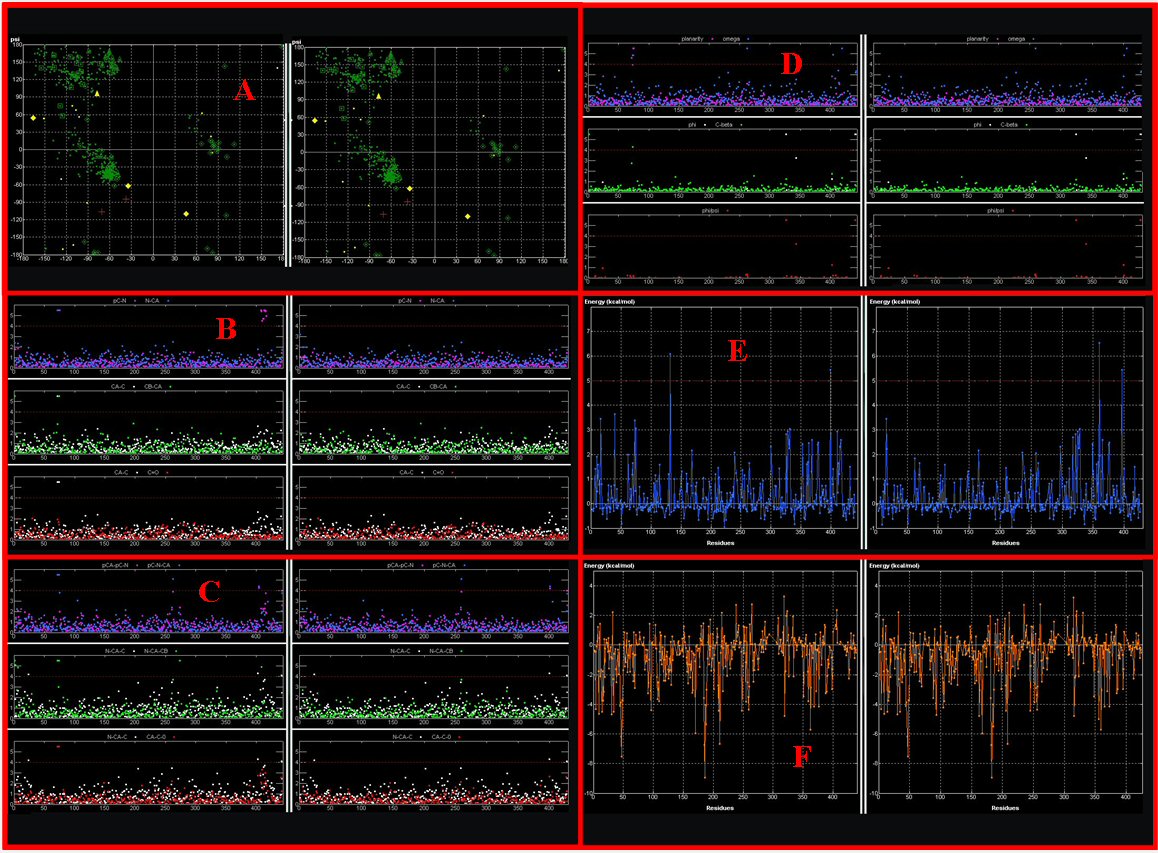

Supplement: Additional file 1 — Extended Procheck Results for both the Usutu virus helicase model and the Murray Valley Encephalitis virus helicase template. Extended Procheck Results for both the Usutu virus helicase model (LEFT COLUMN) and the Murray Valley Encephalitis virus helicase template (RIGHT COLUMN, X-ray structure: 2V80). A: Ramachandran plot, B: Bond Length Plot, C: Bond Angles plot, D: Dihedrals plot, E: Rotamers plot and F: Contact Energies Plot. It is therefore concluded that the Usutu virus helicase model has inherited all structural characteristics of its Murray Valley Encephalitis virus helicase template. [file 1742-4682-6-9-S1.jpeg]
